# Real-time and non-invasive monitoring of plant signaling by means of optical coherence tomography

**DOI:** 10.3389/fpls.2025.1702810

**Published:** 2025-11-21

**Authors:** Adrien Alexis Paul Chauvet, Stephen J. Matcher

**Affiliations:** 1 School of Mathematics and Physical Sciences, The University of Sheffield, Sheffield, United Kingdom; 2 School of Electrical and Electronic Engineering, The University of Sheffield, Sheffield, United Kingdom

**Keywords:** slow wave potential, plant, optical coherence tomography, signaling, rigid registration

## Abstract

This work demonstrates the use of optical coherence tomography (OCT) for studying a plant’s long-range signaling in real time, *in vivo*, and non-invasively. This feat is achieved using OCT as a novel technique to visualize minute cellular displacements and deformations within the plant’s leaves. The use of bespoke registration algorithms enables tracking displacements with a precision greater than 0.1 μm. This measurement precision is one order of magnitude better than the typical ~1-μm optical resolution of OCT images. In the present work, OCT is used to analyze the time evolution of deformations incurred by wounding. The use of OCT enabled to 1) visualize, in real time, the propagation and evolution of the morphological changes associated with slow wave potentials (onset, peak, and recovery); 2) compute propagation speeds (~0.07 cm s^−1^); and 3) distinguish the type of deformation incurred (transient bending of the leaf due to changes in turgor cell pressure). This proof-of-concept study thus exemplifies the potential of OCT as a convenient and complementary tool to study the plant’s response mechanisms *in vivo* and in real time.

## 1 Introduction

To visualize the microscopic structure of plants *in vivo*, non-invasively, and in real time is key to unlock transformative development in botany. Optical coherence tomography (OCT) has all the characteristics necessary to achieve this feat: OCT is an imaging technique that is non-destructive and non-invasive and provides micrometer-resolution cross sections of living tissues in real time (Bouma et al., 2022). OCT thus provides a three-dimensional visualization of the internal structure of plants without the need for histological preparation. Interestingly, while the technique is commonly used in medical fields, and in ophthalmology primarily (Schuman et al., 2024), it is seldom used in the field of botany (Saleah et al., 2024; Sasi and Chauvet, 2025).

**Figure 1:**
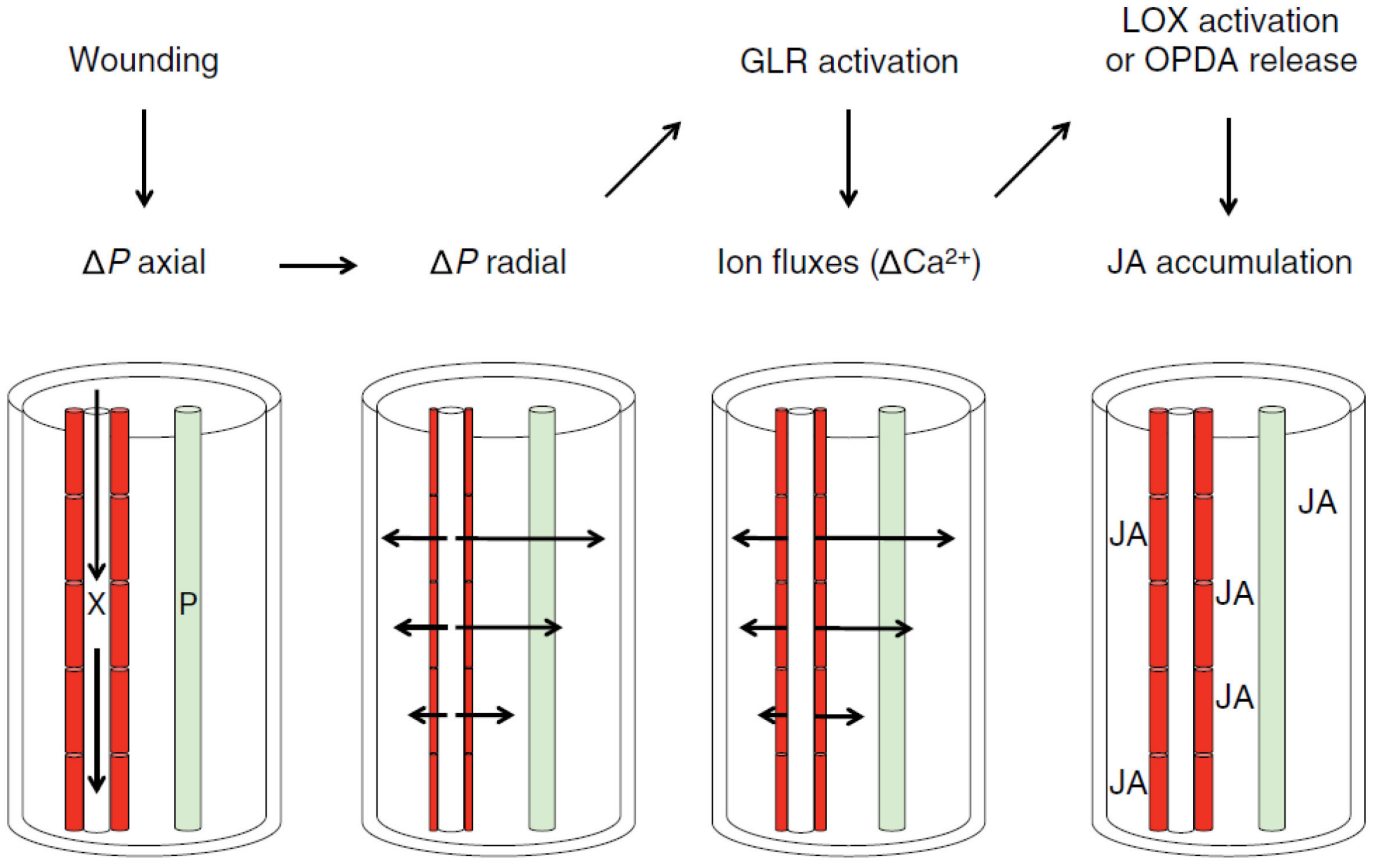
The squeeze cell hypothesis within the bundle sheath. This hypothesis predicts that xylem-transmitted pressure changes generated in wounded plants act in a clade 3 GLR-dependent mechanism that stimulates vascular jasmonate synthesis distal to the wounds. In response to wounding, axial pressure changes are propagated rapidly along xylem vessels (X), and these are then converted to slower radial pressure changes that squeeze xylem contact cells (red). This directly or indirectly modulates glutamate receptor-like (GLR) protein activity, leading to ion fluxes that are propagated in part through plasmodesmata and to the activation of jasmonic acid (JA) synthesis in and beyond contact cells. It is also possible that Ca^2+^ fluxes help to coordinate jasmonate synthesis in cells associated with both the xylem and phloem (P). The potential mechanisms by which GLRs and/or calcium activate jasmonate accumulation include lipoxygenase (LOX) activation or oxo-phytodienoic acid (OPDA) release, as discussed in the text. Wound-induced electrical signaling along the phloem and possible axial jasmonate transport along X or P are not indicated. This figure is an authorized reproduction from Farmer et al (Farmer et al., 2014).

In this work, OCT is used to visualize the morphological changes of a plant when subjected to stressors. More specifically, OCT is used to monitor the minute displacements and changes in leaf morphology as a response to physical damage. This proof-of-concept work fits within a larger scientific enquiry whose aim is to investigate systemic signaling within plants in response to abiotic stressors (Fichman and Mittler, 2021; Johns et al., 2021; Mudrilov et al., 2021; Lee and Seo, 2022; Costa et al., 2023). This work addresses the so-called “squeeze cell hypothesis” proposed by Prof. Farmer (Farmer et al., 2014). Following this hypothesis, wounds inflicted on the plant (e.g., a caterpillar eating leaves) trigger a chain reaction that leads to the secretion of hormones (jasmonates, among others) capable of defending the plant against the stressor (e.g., debilitating the digestive system of caterpillars) (Chen et al., 2005; Johns et al., 2021; Lee and Seo, 2022). In this hypothesis, the production of jasmonates is linked to mechanosensitive anion channels involved in wound signaling (Moe-Lange et al., 2021). As depicted in **Figure 1**, a wound consisting of the piercing of a xylem vessel induces a pressure wave that rapidly propagates throughout the xylem network of the plant. This primary axial pressure wave along the xylem is followed by a secondary radial change in pressure during which the xylem tracheary elements are squeezed. This squeezing opens ion channels, which in turn trigger the production of jasmonates. Here, we are interested in using OCT to monitor the morphological changes implied in this hypothesis. The translation of the latter into jasmonate production and liberation is currently out of scope.

The aim is to monitor the various morphological changes caused by the wound-induced pressure changes. This study thus contributes to the broader endeavor to study signaling in plants. Plants are known to respond to stimuli via three interconnected signaling types: electrical (action potentials, slow wave potentials, and system potentials), hydraulic (changes in turgor pressure, pressure waves, and mass flow), and chemical (reactive oxygen species, ion flow, volatiles, etc.). The squeeze cell hypothesis is one of these interconnected long-range signaling pathways involving at least two pathways: hydraulic and chemical. The hydraulic pathway is of particular interest because it implies changes in cell pressures and thus changes in morphologies, which can be picked up by OCT.

Pressure changes in plants were so far monitored either 1) directly by installing a pressure gauge on the plant (Sack and Holbrook, 2006), 2) indirectly via a pressure probe attached to the leaves (Zimmermann et al., 2008; De Swaef et al., 2012; Zimmermann et al., 2013), or 3) indirectly again by monitoring thicknesses via light probes (Nožková et al., 2018; Mudrilov et al., 2024). The first pressure gauge method is advantageous as it provides a direct measurement of pressure. It is, however, not ideal as it requires cutting part of the plant to access the xylem network. The plant is thus damaged before any experiment takes place. The second pressure probe method is certainly less invasive but still requires the probe to be in direct contact with the plant. Indeed, changes in cell pressure in the leaves imply that individual cells swell or contract, which results in overall changes in the leaves’ thickness. However, because the probes are in direct contact with the leaves, the contact can itself act as a stressor. The third method makes use of light, either by monitoring the shadow of the leaf directly via a light curtain or indirectly through the diffraction pattern created by the edge of the plant. The use of light is ideal because it is non-invasive and can achieve a diffraction-limited resolution. However, these techniques require the plant to be firmly held in place with forceps, which can itself act as a stressor. Ideally, such investigations would require truly non-invasive methods to ensure that plants are not affected by the measurement itself, which is what OCT enables. Accordingly, this work explores the use of OCT to monitor the expected changes in leaf displacement and morphology resulting from the systemic pressure changes induced by wounding.

**Figure 2:**
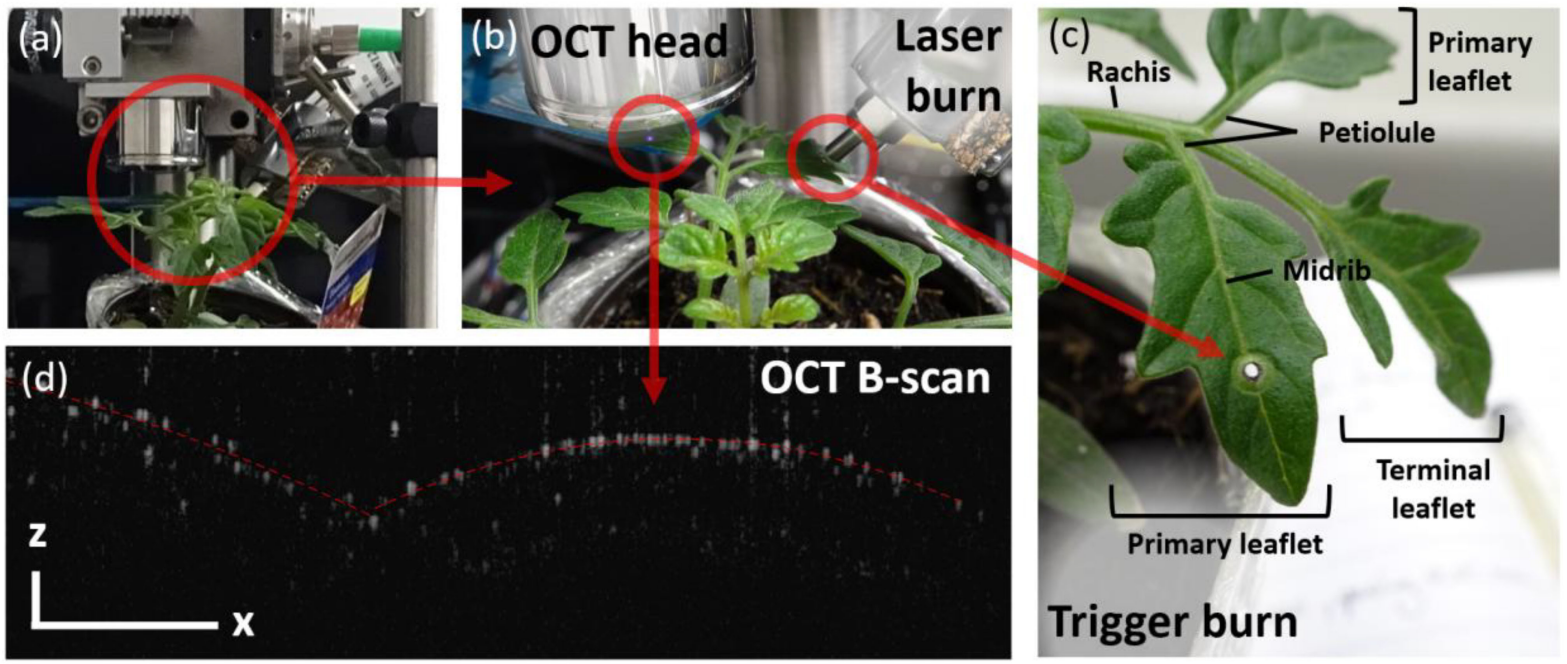
**(a, b)** Experimental setup. **(c)** Leaf’s structure and **(d)** sample OCT B-scan of the leaflet’s cross section. The B-scan is thus characterized by its x- and z-axes, corresponding to the lateral (along the leaf’s surface) and axial (depth) directions, respectively. The scale bar represents 500 μm in each x- and z-direction.

OCT is ideal in this context because the probing consists of an infrared scanning beam: Light is emitted by the scanner head situated a couple of centimeters above the leaf, as shown in **Figure 2**. The light shone onto the leaf is then scattered by the leaf itself, and part of the scattering light is picked up by the same scanner head to be analyzed (Aumann et al., 2019). By analyzing the scattered light, a view of the internal structure of the leaf is generated with a typically diffraction-limited resolution (Wang et al., 2013). In practice, OCT imaging is limited by the optical components of the scanning head, by the density of the sample’s tissues, and by the diode’s central wavelength. Considering all these factors, OCT images commonly have an optical resolution of ~10 μm and up to 1 μm for high-end systems. Soft tissues with air gaps and watery constitution typically allow for optimum resolution when using near-infrared light sources (Zhang et al., 2016). Plants have, however, a huge variability in terms of cell packing and cell density (Lehmeier et al., 2017). For example, while OCT can see through the entire *Arabidopsis’* soft leaves (de Wit et al., 2020), it scarcely resolves the first few cell layers in the sturdy *Triticum* (i.e., wheat) (Vodeneev et al., 2012).

For this proof-of-concept study, we chose a plant that is known to have a strong chemo-electric response, such as *Solanum lycopersicum*, i.e., tomato plant (Alarcon and Malone, 1994; Volkov and Shtessel, 2018). Indeed, besides being relevant to global agriculture (Costa and Heuvelink, 2005), tomato plants have been reported to have some of the strongest systemic responses to wounds (Bowles, 1998). Tomato leaves are, however, quite sturdy (Verboven et al., 2015) and have limited penetration depth at 890 nm (as seen in **Figure 2**). The resulting OCT images are thus challenging because there are only a few distinguishable structural elements besides the leaf’s surface. We therefore do not monitor the leaf’s thickness as it is commonly done in the literature (Wit et al., 2020; Takahashi and Begzsuren, 2022). Fortunately, distinguishing the leaf’s surface is sufficient to monitor the expected displacements and changes in the leaf’s overall morphology, which are the focus of the current study. Although the experiment has been repeated multiple times, we only showcase the result and analysis of one such experimental run. The different experiments were performed on four different varieties, at differing locations on the plants, as discussed in the SI. After each trigger, the plants systematically exhibit a “jerk” response, which can be correlated to slow wave potentials, as determined by the calculated propagation speeds. We here showcase the clearest example of this proof-of-concept experiment, using an initially intact tomato plant.

## 2 Results

### 2.1 Monitoring of the plant via OCT

The tomato plant is young, and the leaves are relatively sturdy. The penetration depth of the 890-nm light is limited to approximately 300 μm only, probably due to the absence of significant scattering elements below the first cell layer. This means that the OCT signal mostly comes from the upper cell layer of the leaf, as shown in **Figure 2**. The fact that most OCT signals originate from the first cell layer simplifies the tracking of the leaflet’s position. Assuming that the leaf’s cells are juxtaposed and are not expected to break apart, the monitored surface is representative of the entire leaf’s motion and deformation. Position tracking is performed via bespoke rigid registration algorithms. Rigid registration is thus used to track the expected leaf’s displacement triggered by a wound.

### 2.2 Monitoring wound-induced responses

The wound consists of a hole burnt by an 800-nm laser through an adjacent leaf (as shown in **Figures 2B, C**). The hole is here used to mimic the damage that a caterpillar would inflict upon munching the leaf. Using light in both cases, to inflict a wound and to monitor the plant’s response, ensures minimal material interference with the plant. In both cases, a near-IR light is selected to guarantee that no photosynthetic-related processes are triggered (Geiger, 1994).

**Figure 3:**
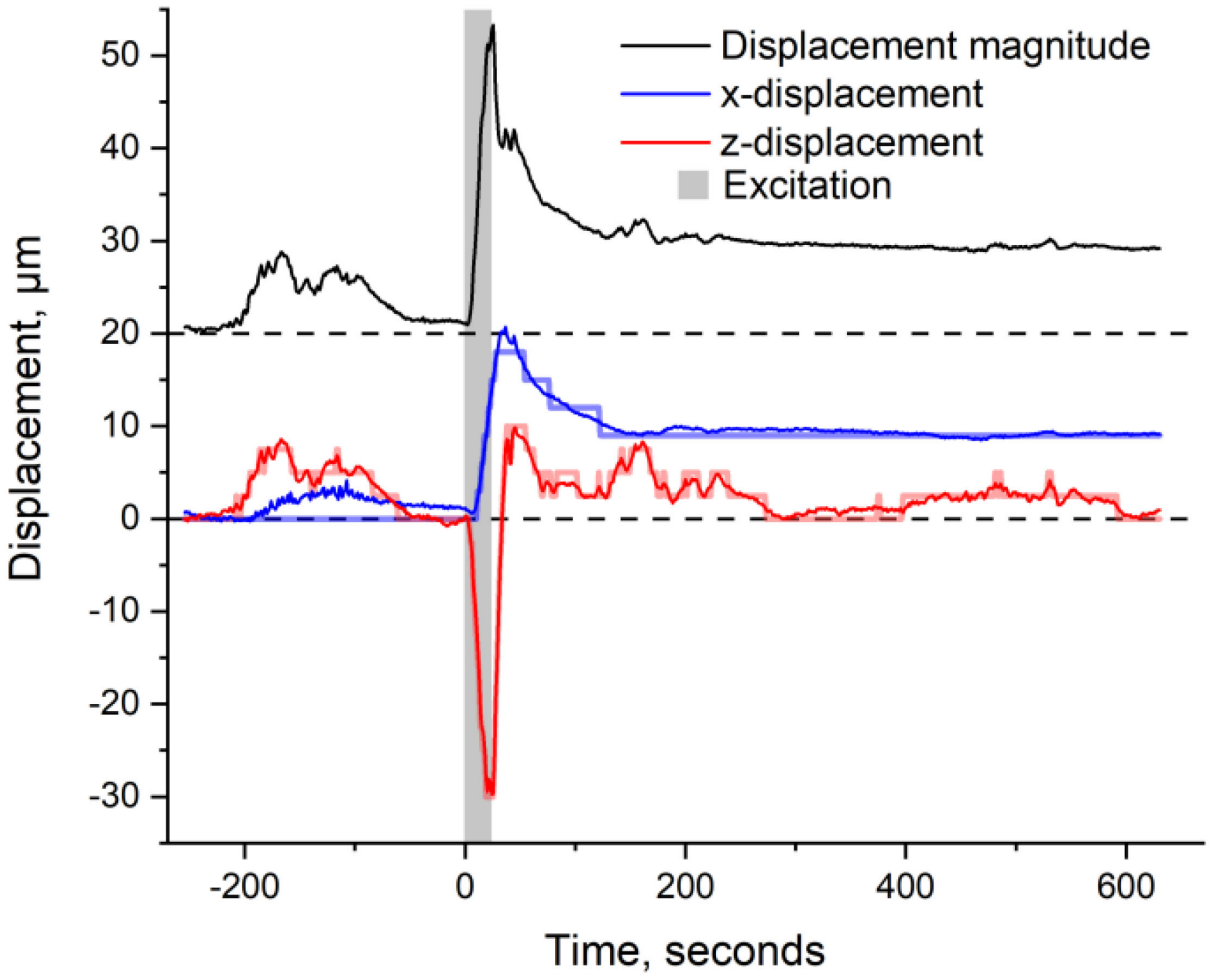
Subpixel registration results showing the leaflet’s horizontal displacement (blue), vertical displacement (red), and magnitude of displacement (black, offset by 20 μm for clarity). The gray-shaded area corresponds to the excitation (e.g., ~20 s of laser burn). The initial full-pixel registration is shown in transparency for comparison (blue and red step-like signal). Negative values correspond to the upper and right-sided motion of the leaflet.

During the experiment, the leaf is continuously monitored via OCT at a rate of 0.8 Hz at a specific fixed location. The wounding starts at *t* = 0 and lasts for about 20 s to ensure that a hole is pierced throughout the leaf’s midrib. This ensures that the wound directly alters the plant’s xylem network. The OCT images are then rigidly registered (i.e., only accounting for x- and z-displacements). The extracted x- (along the leaf’s surface) and z-displacements (perpendicular to the leaf’s surface), resulting from the registration, as well as the magnitude of displacement, are shown in **Figure 3**.

In comparison to the signals here monitored, a morphological change of ~20 μm has been previously monitored in wheat in response to wounding (Vodeneev et al., 2012). Hence, given that our signal includes all, x- and z-translation, as well as deformation of the leaf, the monitored values of ~30 μm are in the expected range. Note that the “displacement magnitude” corresponds to the root mean square (rms) value of the x- and y-displacements (i.e., $\sqrt{\Delta x^{2}+\Delta z^{2}}$).

### 2.3 Rigid registration analysis of the leaf’s response

Using topographical images to monitor the leaf’s displacement has already been demonstrated (Vodeneev et al., 2012; Williams et al., 2020). This work, however, innovates by using a systematic and enhanced registration analysis to monitor submicrometric displacements. The registration analysis is done in two distinct ways: an initial full-pixel registration and a subsequent subpixel registration. The full-pixel registration, shown as the step-like function in **Figure 3**, takes into account the whole of the B-scans but is restricted to a minimal displacement of 1 pixel. The precision of this registration is thus comparable to the size of a pixel, which is ~2.6 μm axially and ~8 μm laterally. In comparison, a subpixel registration is performed on the x- and z-projections of each B-scan. The projections are then interpolated by a factor of 100 and compared independently using a generic minimizing function (from MATLAB), thus improving the registration precision by an equal factor (×100). The subpixel registration is shown by the solid curves in **Figure 3**. Both registration methods yield matching results. This correspondence validates the use of subpixel registration and enables tracking of the leaflet with an unprecedented precision of <0.1 μm. Note that this subpixel registration exceeds by a factor of 10, which is the resolution of monitored morphological changes that Malone’s innovative use of transducers achieves when measuring relative leaf thicknesses (Malone, 1992; Malone, 1993).

## 3 Discussion

**Figure 3** shows that even before excitation (*t* < 0 s), the leaflet jiggles slightly by approximately ±5 μm. This background motion confirms that the plant is alive and that the leaflet is relatively free to move. Upon excitation (*t* = 0 s), the leaflet jerks significantly by ~35 μm from its initial position and relaxes afterward. Note that while the leaflet’s vertical y-position goes back to its original value, the horizontal x-position does not. Partial recovery of the x-position is most probably the result of friction between the leaflet and the support plate, which can be seen in **Figures 2A, B** (blue ruler plate underneath the scanned leaflet).

Since the trigger burn is expected to disrupt the plant’s xylem network, the initial jerk of the leaflet is assigned to a change in the turgor pressure of the cells constituting the rachis (Ye et al., 2008). Indeed, deformation of these cells is expected to result in direct x- and z-displacements of the monitored leaflet. With a maximum displacement occurring 25 s after the start of the excitation and the rachis being situated ~1.5 cm away from the burnt hole, we deduce a minimal signal propagation speed of ~0.06 cm s^−1^. It is important to note that this signaling speed is computed using the time when maximum displacement occurs. A partial displacement is, however, monitored as soon as the 20-s-long wounding starts. This partial displacement could thus be indicative of an immediate response from the plant. This fast response, which takes place during the wounding itself, is similar to the one previously monitored in wounded *Arabidopsis*, but absent in wounded *Mimosa pudica* (Kurenda et al., 2019). Such a variation in response further emphasizes the plant-specific dependence of the “immediate” response to wounding. This fast response will be subject to future work.

**Figure 4:**
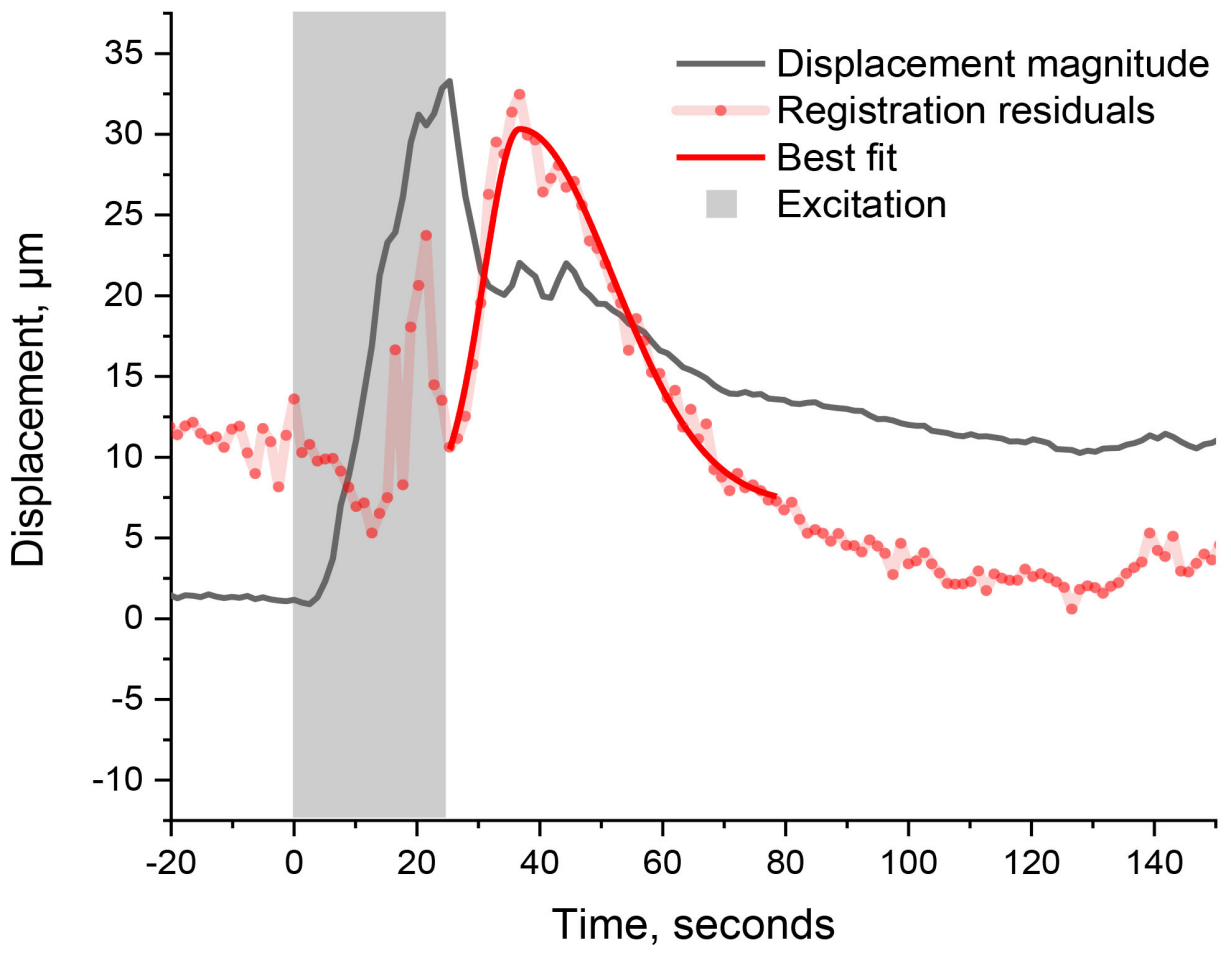
Time-varying variance (red dots, scaled and vertically shifted for convenience) and its best fit using a bi-Gaussian (solid red curve). The leaflet’s displacement magnitude is shown for comparison (black). The gray-shaded area represents the duration of excitation. The sharp signal in the variance curve (outside excitation) corresponds to the expected leaflet’s deformation.

Upon rigid registration, the difference between two images is expected to be minimal when both the image and the reference are superimposed. Accordingly, the variance (i.e., the sum of absolute values of the difference image’s residual pixels) is expected to be minimal. Any deformation of the leaflet would, however, generate some mismatch that regular x, z-registration cannot fully compensate for, and the variance is expected to increase accordingly. The time-varying variance thus becomes an indicator for morphological changes within the leaf, as shown in **Figure 4**.

**Figure 5:**
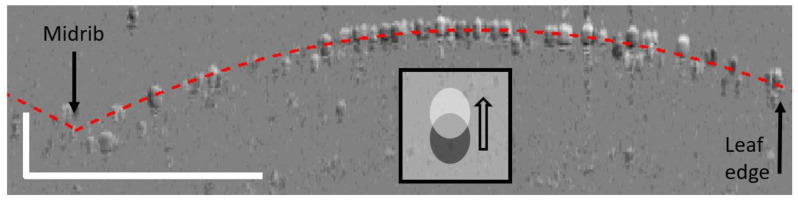
Difference image obtained by subtracting the OCT image taken at 46 s after excitation from the OCT image taken at 9 s before excitation, after registration. The leaflet is marked by some registration mismatch with light-shaded cells above and dark-shaded cells below the red dotted line, which correspond to the surface of the leaf. In such a difference image, cell displacements are shown as going from dark to light regions, as depicted in the inset. The scale bar represents 500 μm in each axis.

In comparison to the magnitude of displacement, the variance has its maximum at 38 s after the wounding starts, which is 13 s after the maximum displacement. Since the OCT-monitored area is situated ~2.7 cm away from the trigger burn, the monitored deformation was caused by a signal travelling at a speed of ~0.07 cm s^−1^. Since this signal speed is comparable to the one computed earlier, it is safe to assume that we are here monitoring the same signaling process, also triggered by the wound, while it travels through two different locations: first when it reaches the rachis (thus moving the whole leaf) and second when it reaches the scanned section on the leaflet (thus deforming the leaflet). Such signal speed coincides with the changes in cell turgor pressure as reported in hydraulic signaling by Huber et al (Huber and Bauerle, 2016). These hydraulic signals are typically associated with the propagation of the slow wave potential (SWP) that precedes cellular depolarization (Stahlberg et al., 2006). Note that the sharp spikes in registration residuals (**Figure 5**, red dots) appearing during the trigger burn (in the gray-shaded region) are artifacts coinciding with the extra scattered light from the trigger burn (as discussed in the SI). Furthermore, given our acquisition rate of 0.8 Hz, we would not monitor any signaling resulting in morphological changes that do not persist longer than 1.25 s. The present analysis is only concerned with the most evident features, thus leaving aside smaller ones, such as the onset of displacement and fluctuation during recovery, for future studies.

To reveal the type of deformation monitored, it is necessary to further analyze the raw images acquired. A typical difference image is obtained when subtracting an OCT image acquired at maximum variance from one acquired before wounding, as shown in **Figure 5**.

The difference image shows that the monitored deformation corresponds to minute displacements of the cells, mostly situated in the upper part of the leaflet rather than those on the edges (**Figure 5**, right) or near the midrib (**Figure 5**, left). These displacements thus correspond to changes in the leaflet’s curvature and/or torsion. Such deformations are illustrative of changes in turgor pressure of the cells underneath the surface. It is indeed reported that, upon wounding with heat, the thickness of neighboring leaves increases due to the intake of sap fluid (Malone et al., 1994). It is important to note that the monitored deformation is occurring aside from the midrib, which is where the main xylem channel passes. We are thus observing the expected deformation of cellular structures neighboring the primary xylem network. This reinforced the conclusion that we are here witnessing the radial pressure changes as they propagate away from the main xylem channels (Stahlberg and Cosgrove, 1997). Such pressure changes, induced by the SWPs, are expected to open the ion channels themselves, resulting in the ultimate production of jasmonate derivatives, as per the “squeeze cell hypothesis.” (Farmer et al., 2014).

In conclusion, this work demonstrates the feasibility of studying long-range signaling in plants, in real time, *in vivo*, and non-invasively. This feat is achieved using OCT as a novel technique to monitor minute cellular displacements and deformations. Although OCT often suffers from limited optical resolution (~1 μm) and lower penetration depth (<1 mm), compared with the usual confocal or light-sheet microscopies, the use of bespoke registration algorithms enables tracking of displacements with a precision greater than 0.1 μm. In the present work, OCT was used to monitor the time evolution of deformations incurred by wounding. More specifically, the use of OCT permitted the visualization, in real time, of the morphological changes associated with the propagation of SWPs involved in the “squeeze cell hypothesis,” to compute propagation speeds, and to distinguish the type of deformation incurred. This study thus demonstrates that OCT is an ideal tool to study plants’ signaling pathways. More generally, this study opens the door to live monitoring of plants’ responses to biotic and abiotic stressors. Paving the way for live imaging is of particular importance in stress management in horticulture as well as in crop monitoring.

## 4 Methods

### 4.1 The plant

The tomato plant (*Solanum lycopersicum*) used in this experiment is a Sweet Million variety, circa 4 weeks old, acquired from the local nursery. The plant was kept in individual pots, at room temperature, with ambient lighting and low humidity (<40%) conditions. It is important to note that, while the pot is mounted directly on the stabilized laser table, the plant is not held during the experiment. As shown in **Figures 2A, B**, the monitored leaf “sits” on a fixed (blue) plate to ensure its surface is perpendicular to the laser beam. The technical scheme of the experimental setup is given in the **Supplementary Information (SI)**. The whole plant is enclosed to protect the user against possible scattering from the laser burn and to minimize vibrations from interfering air currents. Consequently, the leaf is relatively free to move, as demonstrated by the slow “breathing” motions of the plant at negative times in **Figure 3**.

### 4.2 The wound

The wound consists of a laser burn. The burn is done by focusing a 6-W, 800-nm laser onto one of the primary leaflet’s midribs (see **Figure 2**) for approximately 20 s. At this power and wavelength, it takes about 10 s to burn a hole through the leaf. The midrib is targeted to ensure the perforation of primary xylem vessels, which is expected to yield maximum systemic response. Because the laser is turned on for 20 s only, and because the end fiber is located a few millimeters away from the leaf’s surface, the ambient heat produced by the laser is not expected to create any air convection, which could have influenced the measurement. Because we cannot ensure that the plant responds similarly to subsequent stimulus, the experimental run is performed on a new plant each time. The data presented in this work are thus from an initially intact plant.

### 4.3 OCT setup

The technical details of the OCT system have been previously published (Boadi et al., 2015). In brief, the system is a custom-built ultrahigh optical resolution spectral domain OCT. The ~890-nm light from a dual superluminescent diode source is split into reference and sample beams. The sample beam is deflected by an x–z galvo pair and focused by a telecentric OCT scan lens with an effective focal length of 18 mm. The reference arm consists of a fixed plane mirror, an adjustable neutral density filter, and a dispersion compensator. The recombined sample and reference beam are detected by a spectrometer constructed in-house. A-scans (depth profiles) were acquired at a rate of 20 kHz, and B-scans were assembled, consisting of 1,000 A-scans. After subtraction of the d.c. component, fringes were resampled using a technique based on detecting the fringe zero-crossings and using the interpolated pixel positions of these to build a non-linear interpolation table between pixel value and *k*. Pixels outside the interference spectrum are zeroed. Following these steps, the system has a measured axial resolution in air of 2.6 µm. The lateral resolution was measured using the USAF resolution target and found to be 8 µm. Although the images acquired in this specific study are marked by poor penetration depth (~300 µm), we benefit from this high optical resolution to monitor the leaf’s surface and thus position. With a plane mirror as the sample, a sensitivity of 93 dB was achieved. The system automatically adjusts the image’s contrast range to subtract the contribution from ambient lighting.

The OCT’s scanning objective is positioned at 1.8 ± 2 cm above the (non-held) leaflet adjacent to that where the trigger burn is induced, as shown in **Figure 2**. The system is set to automatically average every 25 B-scans to a target output rate of 0.8 Hz. Images are continuously acquired and individually saved for the duration of the experiment.

### 4.4 Leaf surface detection

Given the limited penetration of the 890-nm light, only the epidermis is expected to give maximum contrast (i.e., little scattering is expected to be detected from the cells situated underneath). The surface of the leaf is thus determined by the position of that maximum pixel value. Because the leaf is horizontal and spans the whole x-axis of the B-scan, we expect a single pixel maximum for each constituting A-scan (i.e., vertical pixel column of the image). Artifacts, however, arise when the light, coming from above the leaf, is scattered by a trichome before it reaches the leaf’s epidermis. These artifacts give rise to spikes on the modeled surface. These artifacts are dealt with by assuming that the number and diameter of trichomes are relatively small compared to the overall leaf’s surface. They are eliminated by smoothing the modeled surface using MATLAB’s *smoothdata* function. The upper surface of the leaflet is then approximated as a double parabola, with each section of parabola corresponding to the left and right sides of the leaflet, and the two sections of parabola meeting at the central midrib. The modeled surface only serves as a visual cue and is represented by the red dotted line on the B-scans (**Figure 2D**) and B-scan difference images (**Figure 5**). Because smoothing is used to generate a visual cue and is not used for the actual analysis, it has no repercussions on the conclusions.

### 4.5 Image registration

The leaflet’s displacement is tracked using two different bespoke registration algorithms:

1) Full-pixel registration, which takes into account the whole B-scans. Each B-scan corresponds to a cross section of the leaf. This x–z cross section is then continuously monitored (the same location) over the duration of the experiment. It is worth emphasizing that we are only analyzing B-scans and not full volumetric C-scans, since the location on the z-axis remains identical throughout. The registration is performed by comparing every B-scan to a reference (called A). The difference between an image (called B) at a specific time delay and the reference (A) yields a difference image (B−A). The image (B) is then translated vertically (z) and horizontally (x) to minimize the variance (i.e., sum of absolute values of the difference image’s residual pixels, $\sum_{x,z}\sqrt{{\left(\text{B}-\text{A}\right)}^{2}}$) using MATLAB’s *patternsearch* minimizing function with default mesh size. This process yields a rigid registration with a precision of 1 pixel.
2) Subpixel registration, which only takes into account the image’s vertical and horizontal projections. In this case, the registration is performed by comparing the x- and z-projections of every image to a reference. The x- and z-projections are then shifted independently to minimize the variance. Because the minimization is performed by comparing single projection vectors (instead of whole images), the vectors (i.e., x- and z-projections) can be readily splined to achieve a subpixel correspondence. The algorithm uses the same MATLAB’s *patternsearch* minimizing function. The interpolation is done using MATLAB’s cubic *spline* function, with its default settings. Interpolating the projection vectors by a ratio of 100:1 yields a rigid registration with a precision of 0.01 pixel. Interpolating by a higher value would not lead to higher measurement precision, as we would be overfitting the speckle noise of the images without improving in x- and y-position precision. The interpolation ratio of 100:1 seems to give the best results in our case, as demonstrated in the SI. Although the step size is effective at 26 nm, the precision achieved is estimated at 0.1 µm, as discussed in the SI.

### 4.6 Registration analysis

Once registered, the x- and z-displacements resulting from the rigid registration can either be analyzed separately, as in **Figure 3**, or combined into a single displacement magnitude value ( $\sqrt{\Delta x^{2}+\Delta z^{2}}$), and compared to the variance, as in **Figure 4**. While the x- and z-displacements represent horizontal and vertical motions of the leaf, an increase in variance corresponds to deformations that are not compensated via rigid registration, such as rotation and deformation of the leaf (bending, shrinking, etc.).

## Data Availability

The raw data supporting the conclusions of this article will be made available by the authors, without undue reservation.
