# Supplementary material for: Real-time and non-invasive monitoring of plant signaling by means of optical coherence tomography
Source: Front Plant Sci. 2025 Nov 21;16:1702810. doi: 10.3389/fpls.2025.1702810 (PMC12678304; doi:10.3389/fpls.2025.1702810)
Supplement: Supplementary file 1 [file DataSheet1.pdf]

Real-Time and Non-Invasive Monitoring of Plant Signaling by Means of Optical Coherence Tomography

Adrien Alexis Paul Chauvet [1,*] and Stephen Matcher [2]

1 School of Mathematics and Physical Sciences, The University of Sheffield, Dainton Building, Sheffield, S3 7HF (UK)
2 School of Electrical and Electronic Engineering, The University of Sheffield, 3 Solly Street, Sheffield, S1 4DE (UK)
* Correspondence: author: a.chauvet@sheffield.ac.uk

Supplementary Information

1. Experimental setup

During the experiments, although the pot is firmly maintained on the optic laser table, the plant itself is left relatively free to avoid impeding its response to the laser burn. Only the monitored leaf sits on a plate to ensure that the leaf's surface remain approximatly perpendicular to the OCT light source, as shown in Figure S 1.

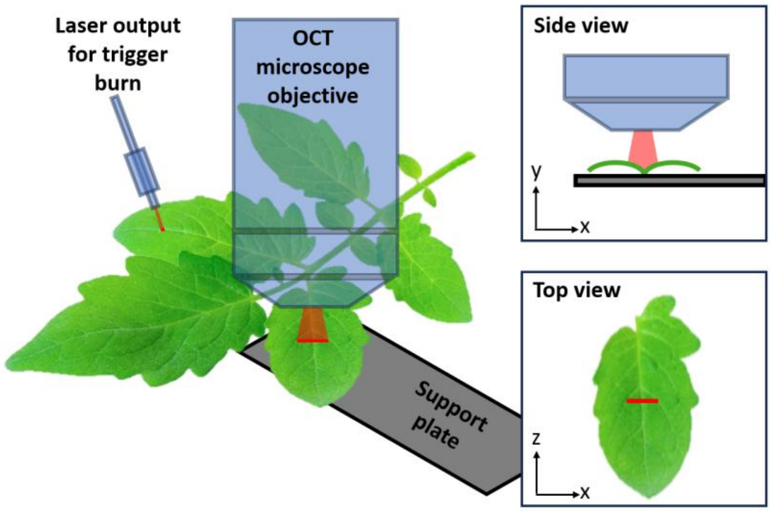

Figure S 1: Scheme of the experimental setup with side and top views in insets.

The leaf is thus free to move along the x-axis (left and right), and partially along the z-axis (upward and minimally downward). Because the B-scan is perpendicalar to the leaf's midrib, it is not expected to move in the y-direction. The potential y-motions can however be estimated by looking at subsequent B-scans: because the B-scans exhibit the same cellular features, as shown in Figure S 2, the leaf is not moving beyond a single cell diameter, which is estimated at 20 um laterally.

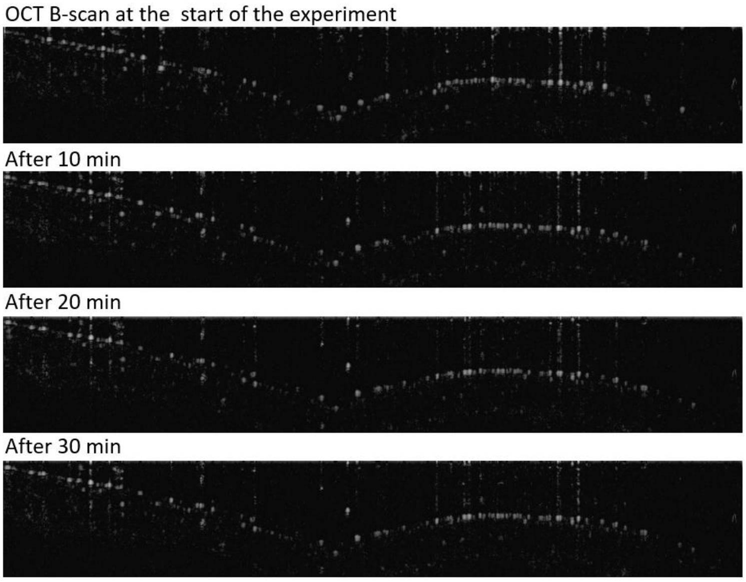

Figure S 2: OCT B-scans at different time points.

We ascribe the poor depth penetration to the waxy cuticle of the leaves. The current variety of tomato (sweet million) is especially sturdy, compared to alternatives, which rendered much greater depth resolution, while using the exact same setup (https://opg.optica.org/abstract.cfm?uri=OCT-2020-OM4E.6).

2. Full and sub-pixel analysis software

The MATLAB code used for analysis are available at the following link:
https://drive.google.com/drive/folders/149Ij2MSEh_yM5kRE_m0EbfVTDppBlCwC?usp=sharing
For enquiries, please contact the corresponding author: Dr Adrien Chauvet, a.chauvet@sheffield.ac.uk

3. Sub-pixel analysis optimisation

The sub-pixel analysis relies on a cubic spline function, which is used to interpolate the x- and z-projections of individual OCT B-scans. No interpolation (i.e. no spline) indicates that the fitting step size is equal to one full pixel (i.e. ~ 2.6 µm). As shown in Figure S 3, there are no x-displacement monitored, between 400 and 600 sec after the trigger burn, with a resolution of +/- ~ 2.6 µm (grey data points).

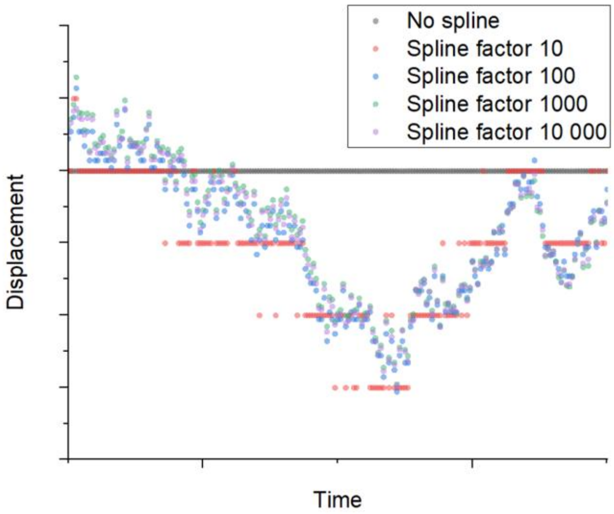

Figure S 3: Zoom of the x-displacement timepoints between 400 and 600 sec after laser burn, when the leaf has regained partial stability, with various spline factors.

When interpolating with a factor 10 (9 interpolated data points between each two-measured values) using Matlab's cubic spline function, we can then discern displacements with a ~ 0.26 µm step size (Figure S 3, red data points). The resolution is further improved when interpolating with a factor of 100 (Figure S 3, blue data points), thus corresponding to a step size of ~ 26 nm.
Subsequent itterations, when interpolating with a factor of 1000 and 10 000 do not lead to a greater resolution (Figure S 3, green and purple data points, respectively). The 1000x and 10 000x interpolations correspond to a step size of ~ 2.6 and ~ 0.26 nm, respectively. The scattered data appear to superimpose, without being restricted to any obvious "step size" (as it is the case for the grey and red data, Figure S 3). From the extent of this scattering (i.e. range of values around which the data varies), we deduce an inherent resolution of ~ 0.1 µm. Hence, it seems that we have reached our optimal resolution with an interpolation factor of 100.

4. Standard deviation of measurements

To assess the vibrations of the plant itself, as suggested, we deemed it more suitable to assess the variations on each x- and z- axis while the leaf is considered immobile, e.g. at times between -50 and -30 seconds before excitation, as shown in Figure S 4.

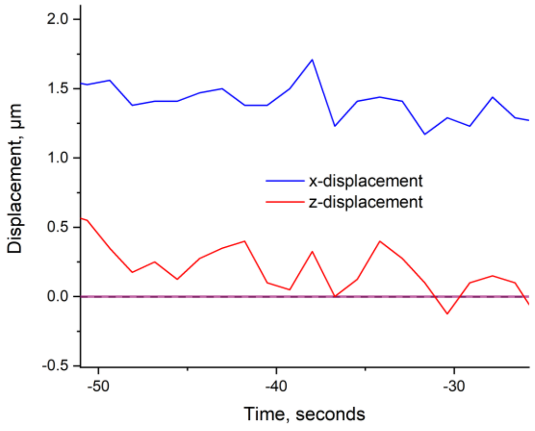

Figure S 4: Sub-pixel registration results showing the leaflet's horizontal displacement (blue) and vertical displacement (red).. This is a close-up of the main manuscript's Figure 3 at times from -50 to -30 seconds, to show minimal displacements when the leaf is considered immobile.

Assuming that the leaf is steady between -50 and -30 seconds before excitation implies that we are here monitoring the inherent vibration of the leaf and/or of the apparatus. Analysing this segment enables to compute a standard deviation of 0.12 and 0.17 µm for the x- and z-axis, respectively, which is in close agreement with the previously estimated 0.1 µm resolution.

5. Impact on leaf and on OCT of the laser trigger burn

The time-varying data, presented in Fig 4, that peaks during the excitation are most probably caused by artifact of the trigger. The trigger consists in turning a 800-nm laser ON for 25 sec while it is focussed on the nearby leaf. The OCT is expectded to detect the extra scattered light emanating rom the trigger.

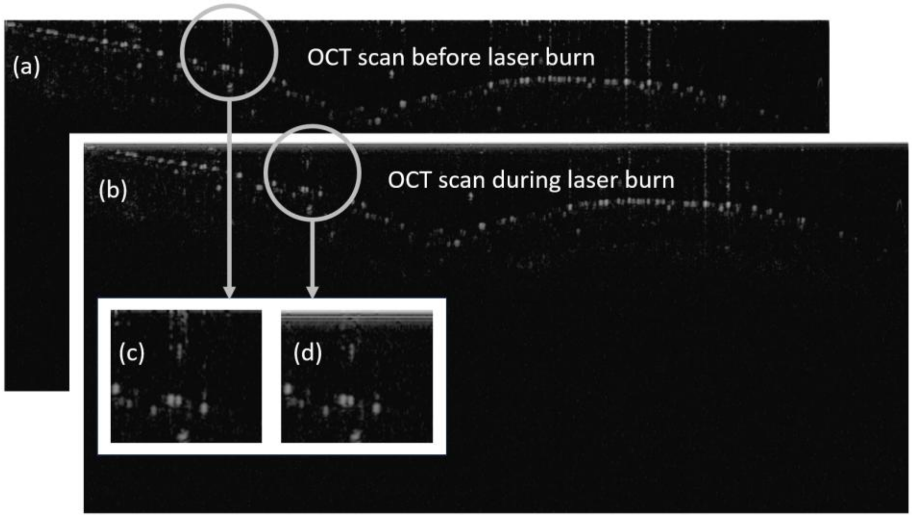

Figure S 5: Comparison between OCT scans taken (a) before the trigger and (b) during the trigger. The inset shows a zoomed section of of each scan, illustrating the extra speckle noise when the laser burn is ON (d) compared to when it is OFF (c).

It typically takes 10 sec to burn a hole through a green leaf. Consequently, after 10 sec, the trigger laser light passes through the leaf. The OCT is equally expected to detect the extra scattering of the laser light passing through the leaf.

Note that heat dissipation via convection, transpiration, diffusion, etc. is expected to be highly ineffective (in comparison to the expected signalling) both in terms of distance and propagation speeds. The fact that a hole is burn within ~10 seconds only of illumination is the proof that heat is accumulated and not efficiently dissipated.

It is actually not straightforward to obtain numerical values for heat dissipation via water in leaves partly due to the complexity of the xylem network and the flow dependence on internal pressure. See

https://academic.oup.com/jxb/article/65/7/1895/542993

https://link.springer.com/article/10.1007/s40415-017-0430-z

Furthermore, because the large displacements (20 µm on the x-axis, 30 µm on the z-axis) coincides temporally with the trigger burn, it is assume that they are directly correlated to the damage incurred.

6. Alternative experimental runs

The same experiemnt was performed on various tomato cultivars:

- Pepper Blondie
- Plum
- Golden Sunrise
- Sweet Million (Experimental run presented in main text)
- Super Sweet 100 (SI-experimental run A and B, below)

The burn and OCT monitoring location were alternatively placed on the same leaf, on adjacent leaflets, or on adjacent leaves. In each case the analysis monitored a "jerk" in response to the laser burn, similar to the one showcased in the main text.
As expected, the intentisty of the monitored response is directly related to the damaged area. Maximum response is optained when the leaf's main xylem vessel is damaged. Burning a whole besides one of the primary xylem vessel results in minimal responses.
The main difference monitored across plant varieties is the variable penetration depth and thus overall OCT image quality.

a) Experimental run A

The setup is similar to the one presented in the main text, as shown in Figure S 6. None of the OCT scanning head nor the fiber optic used to inflict the trigger burn are directly touching the plant. The trigger burn was aimed at a leaflet's midrib to ensure maximal response, depicted in Figure S 7.

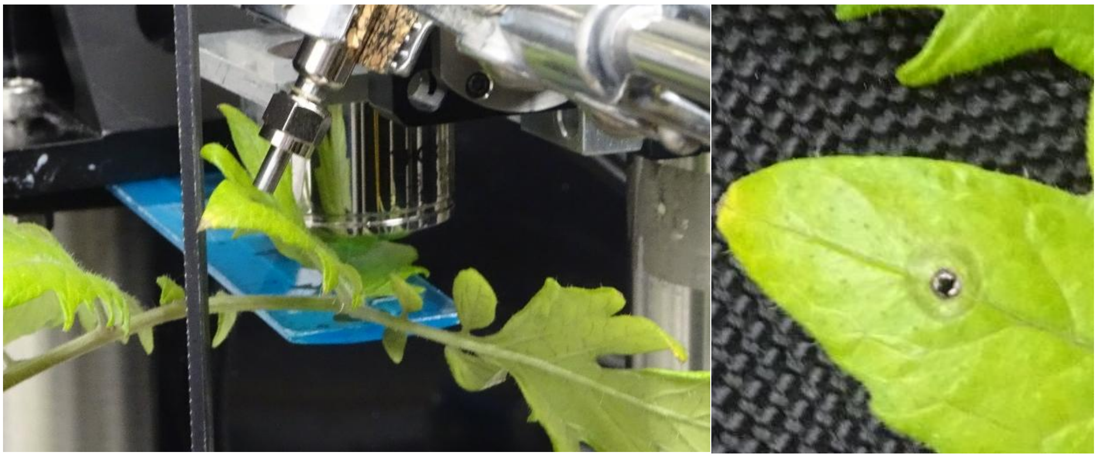

Figure S 6: Left – Side view of experimental design with OCT setup above the leaflet (microscope objective, back) and trigger burn above the adjacent leaflet (optic fiber, front). Right – damaged area induced by the trigger burn.

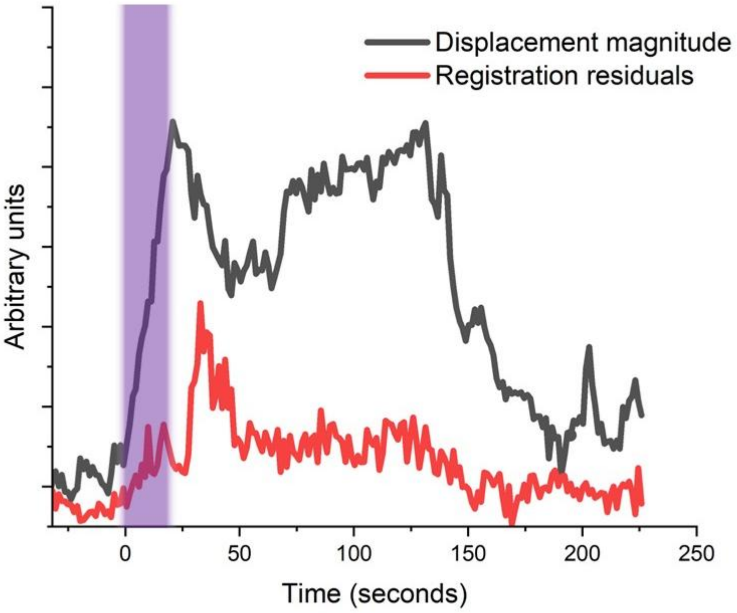

Figure S 7: Magnitude of displacement (black) and residuals of solid registration (red) from the experimental run A. The purple shaded area corresponds to the period during which the trigger laser was ON (~20 sec).

The magnitude of displacement and residuals from solid registration analysis are computed as described in the main text. The maximum displacment is reached 21 sec after the laser for the trigger

burn is being turned ON. Maximum dis-lacment is expected when the signalling wave has reached the rachis (which is expected to induce motion of the whole leaf). Given the distance of 7.5 cm between the laser burn and the rachis, we compute a signalling speed equal to ~0.36 cm s^{-1}.

The maximum in residuals from the sub-pixel registration analysis occures at 33 sec after the start of the excitation. Given that the OCT monitores the surface of the leaflet which is about 13 cm away from the trigger burn, we compute a signalling speed equal to ~0.39 cm s^{-1}. This signalling speed is in good agreement with the above estimate obtained by analysing the magnitude of displacement. Although this signalling speed is five time faster copmared to the one reported in the main text (~0.7 cm s^{-1}), and is potentially due to the fact that we are comparing different tomato varieties, it still coincides to the expected slow wave potential propagation speeds (0.08 – 0.5 cm s^{-1}, 10.1093/jxb/erw099). Note that the registration residual are here exempt from light scattering fron the trigger burn. This is achieved by adequatly croping the OCT images to avoid taking the light interferences into consideration during the analysis.

We note that, similarily to the experimental run preented in the main text, while the deformation (represented by the signal on the registration residual curve) is transient (lasting ~20 sec), the leaf's displacment extens beyon 150 sec after excitation before recovering. We confirm that this longer lasting signal corresponds to a mere translation of the leaf by looking at a OCT different image, shown in Figure S 8.

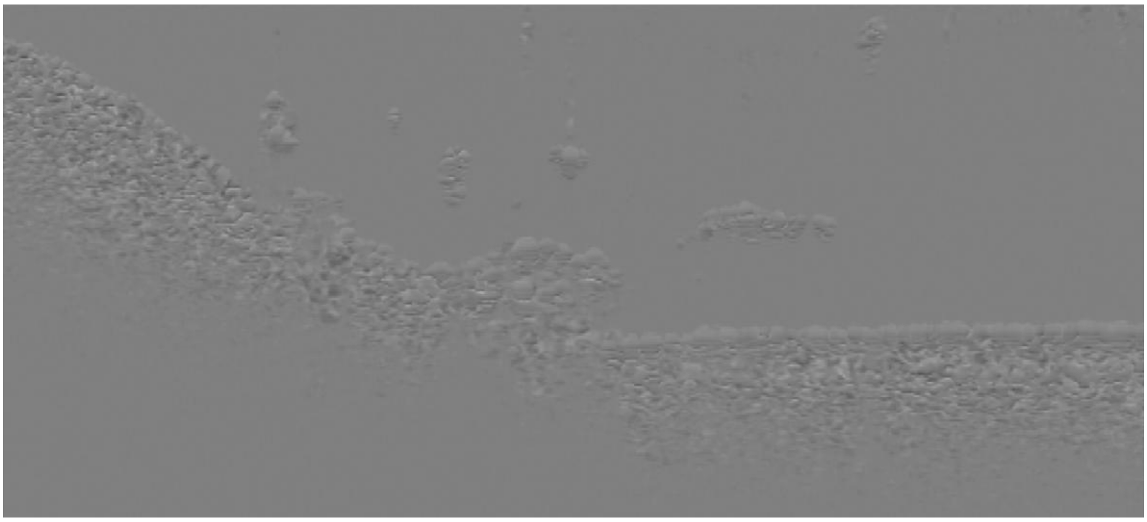

Figure S 8: Difference image between averages of OCT scans before trigger burn and ~100 sec after trigger burn.

By inspection of the difference image depicted in Figure S 8, one can see that all ditinguishable cells have a light-grey upper part and a dark-grey lower part. Shuch colour featur is indicative of a simple leaf's downward translation.

b) Experimental run B

The setup is similar to the one presented in the main text, as shown in Figure S 6. None of the OCT scanning head nor the fiber optic used to inflict the trigger burn are directly touching the plant. The trigger burn was aimed at a the top leaflet's midrib to ensure maximal response, depicted in Figure S 9Figure S 7.

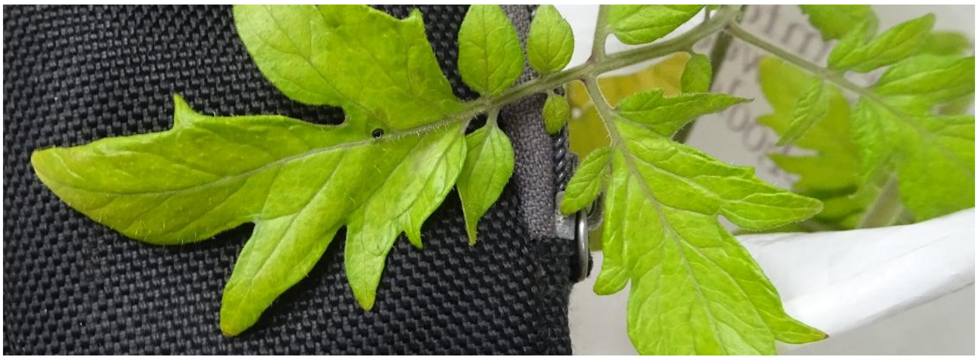

Figure S 9: Same experimental design as in Figure S 6, with the trigger burn located above the terminal leaflet's midrib.

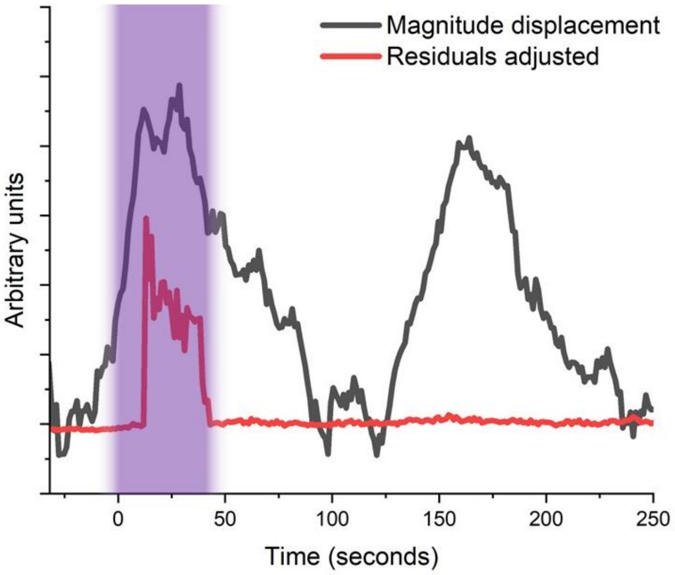

Figure S 10: Magnitude of displacement (black) and residuals of solid registration (red) from the experimental run B. The purple shaded area corresponds to the period during which the trigger laser was ON (~40 sec).

The magnitude of displacement and residuals from solid registration analysis are computed as described in the main text. The maximum displacment is reached 12 sec after the laser for the trigger burn is being turned ON. Maximum dis-lacment is expected when the signalling wave has reached the rachis (which is expected to induce motion of the whole leaf). Given the distance of 4.5 cm between the laser burn and the rachis, we compute a signalling speed equal to ~0.38 cm s^{-1}, thus in agreement with the previously reported signalling speed for slow wave potentials.

In this experiemental run, the registration residuals include considerable light scattering from the trigger burn, starting ~10 sec after the laser is turned ON, which coincide with the time taken by the laser to pierce through the leaf. Again, the amount of scattering included in the data depends on how the OCT images are croped for analysis. Here, the tirgger burn is here kept much longer, beyond the expected transient morphological changes, to demonstrate that these morphological changes are direclty caused by the initiation of the tirgger burn, and not by its termination, as it could have been argued. Looking at these experiments from a transistor logic point of view, this experiment demosntrates that the plant responds to the rising edge of the trigger, and not its decreasing edge.
